# The Formation of the Goldfish-Like Fish Derived From Hybridization of Female Koi Carp × Male Blunt Snout Bream

**DOI:** 10.3389/fgene.2018.00437

**Published:** 2018-10-10

**Authors:** Yude Wang, Conghui Yang, Kaikun Luo, Minghe Zhang, Qinbo Qin, Yangyang Huo, Jia Song, Min Tao, Chun Zhang, Shaojun Liu

**Affiliations:** State Key Laboratory of Developmental Biology of Freshwater Fish, College of Life Sciences, Hunan Normal University, Changsha, China

**Keywords:** distant hybridization, crucian carp, goldfish, microsatellite DNA, 5S rDNA

## Abstract

Goldfish (*Carassius auratus* var., GF; 2*n* = 100) is the most popular ornamental fish in the world. It is assumed that GF evolved from red crucian carp (*C. auratus* red var., RCC; 2*n* = 100). However, this hypothesis lacks direct evidence. Furthermore, our knowledge of the role of hybridization in the formation of new species is sparse. In this study, goldfish-like fish with twin tails (GF-L; 2*n* = 100) was produced by self-mating red crucian carp-like fish (RCC-L; 2*n* = 100) derived from the distant crossing of koi carp (*Cyprinus carpio haematopterus*, KOC; 2*n* = 100; ♀) with blunt snout bream (*Megalobrama amblycephala*, BSB; 2*n* = 48; ♂). The phenotypes and genotypes of GF-L and RCC-L were very similar to those of GF and RCC, respectively. Microsatellite DNA and 5S rDNA analyses revealed that GF-L and RCC-L were closely related to GF and RCC, respectively. The presence of a twin tail of GF-L was related to a base mutation in *chordinA* from G in RCC-L to T in GF-L, indicating that the lineage of RCC-L and GF-L can be used to study gene variation and function. The sequences of 5S rDNA in GF-L and RCC-L were mapped to the genomes of CC and BSB, which revealed that the average similarities of both GF-L and RCC-L to CC were obviously higher than those to BSB, supporting that the genomes of both RCC-L and GF-L were mainly inherited from KOC. GF-L and RCC-L were homodiploids that were mainly derived from the genome of KOC with some DNA fragments from BSB. The reproductive traits of GF-L and RCC-L were quite different from those of their parents, but were the same as those of GF and RCC. RCC-L easily diversified into GF-L, suggesting that RCC and GF evolved within the same period in their evolutionary pathway. This study provided direct evidence of the KOC–RCC–GF evolutionary pathway that was triggered by distant hybridization, which had important significance in evolutionary biology and genetic breeding.

## Introduction

Goldfish (*Carassius auratus* var.,GF; 2*n* = 100) and red crucian carp (*C. auratus red* var., RCC; 2*n* = 100), are the most prevalent ornamental fish in the world, and these species belong to *Cyprinidae* (family), *Cyprininae* (subfamily), and *Carassius* (genus) (Luo et al., 1999; Wang et al., 2014). GF and RCC are considered varieties of crucian carp (*Carassius carassius*). An obvious difference between GF and RCC is that GF has distinct split double tails (twin tail), whereas RCC does not. Although some studies have suggested that GF evolved from crucian carp (Podlesnykh et al., 2015), the direct evidence of its evolutionary pathway is lacking. Hybridization promotes species formation and the adaptive radiation of animals and plants (Mallet, 2007). In plants, some homodiploid hybrid species have been reported, e.g., in *Helianthus* (Rieseberg et al., 1995, 2003; Ungerer et al., 1998), *Vigna* (Takahashi et al., 2015), *Iris* (Arnold et al., 2012), and *Pinus* (Mao and Wang, 2011). There have been few reports on the formation of homoploid in animals; for example, the formation of a homodiploid crucian carp (Wang et al., 2017). Furthermore, our knowledge of the role of hybridization in the formation of new animal species is sparse.

In the catalog, in *Cyprininae* (subfamily), there are only two kinds of species: *Cyprinus carpio* and *C. auratus*, which belong to *Cyprinus* (genus) and *Carassius* (genus), respectively. What is the relationship between these two kinds of species? Both GF and RCC are varieties of *C. auratus*, and most individuals of these species are characterized by red or colorful bodies. Koi carp (*Cyprinus carpio haematopterus*, KOC; 2*n* = 100) is a variety of *Cyprinus carpio*, and most individuals of these species are also characterized by red or colorful bodies. Based on the close status in the catalog and similar body colors among RCC, GF, and KOC, it is possible that GF originate from RCC or KOC by distant hybridization. Blunt snout bream (*Megalobrama amblycephala*, BSB; 2*n* = 48) is a suitable species to cross with KOC. BSB belongs to *Cyprinidae* (family), *Cultrinae* (subfamily), and *Megalobrama* (genus). Compared with KOC, BSB possess different chromosome number (2*n* = 48), different body colors (gray) and the same age of sexual maturity (2 years). In this study, we cross female KOC with male BSB and obtain red crucian carp-like fish (RCC-L) and goldfish-like fish (GF-L), which are homodiploids mainly derived from the genome of KOC with some DNA fragments from BSB, showing the potential of interspecific hybridization to produce new homoploid species in fish.

## Materials and Methods

### Ethics Statement

The procedures were conducted in accordance with the approved guidelines. Experimental fish individuals were housed in open pools (0.067 ha) with suitable pH (7.0–8.5), water temperature (22–24°C), dissolved oxygen content (5.0–8.0 mg/L) and adequate forage at the State Key Laboratory of Developmental Biology of Freshwater Fish, Hunan Normal University, China. The fish used as the samples were anesthetized with 100 mg/L MS-222 (Sigma-Aldrich, St. Louis, MO, United States) before dissection.

### Animals and Crossing Procedure

All samples were cultured at the State Key Laboratory of Developmental Biology of Freshwater Fish, Hunan Normal University, China. The female and male of KOC and BSB reached sexual maturity at 2 years, while the female and male of RCC and GF reached sexual maturity at 1 year. During the reproductive season (April–July) in 2015–2017, 20 mature females and 20 mature males of KOC and BSB were selected as the maternal and paternal parents, respectively. The crosses were performed in two groups: in the first group, KOC and BSB were used as the maternal and paternal parents, respectively; and in the second group, the maternal and paternal parents were reversed. The mature eggs were fertilized with semen, and the embryos were developed in culture dishes at a water temperature of 18–23°C. In the first group, the KOC (♀) × BSB (♂) resulted in two types of offspring: red crucian carp-like fish (RCC-L) and gynogenetic koi carp (GKOC). In the second group, the cross of BSB (♀) × KOC (♂) did not produce any living progeny.

In April, 2016, the male and female RCC-L that reached sexual maturity at 1 year were mated to produce the second generation. In the second generation, there were two types of offspring: red crucian carp-like (RCC-L-F_2_) and goldfish-like fish (GF-L) with split double tails.

In December, 2017, the eggs and the white semen were stripped from the female and male of GF-L, respectively, and they were fertilized to form GF-L-F_2_.

**FIGURE 1:**
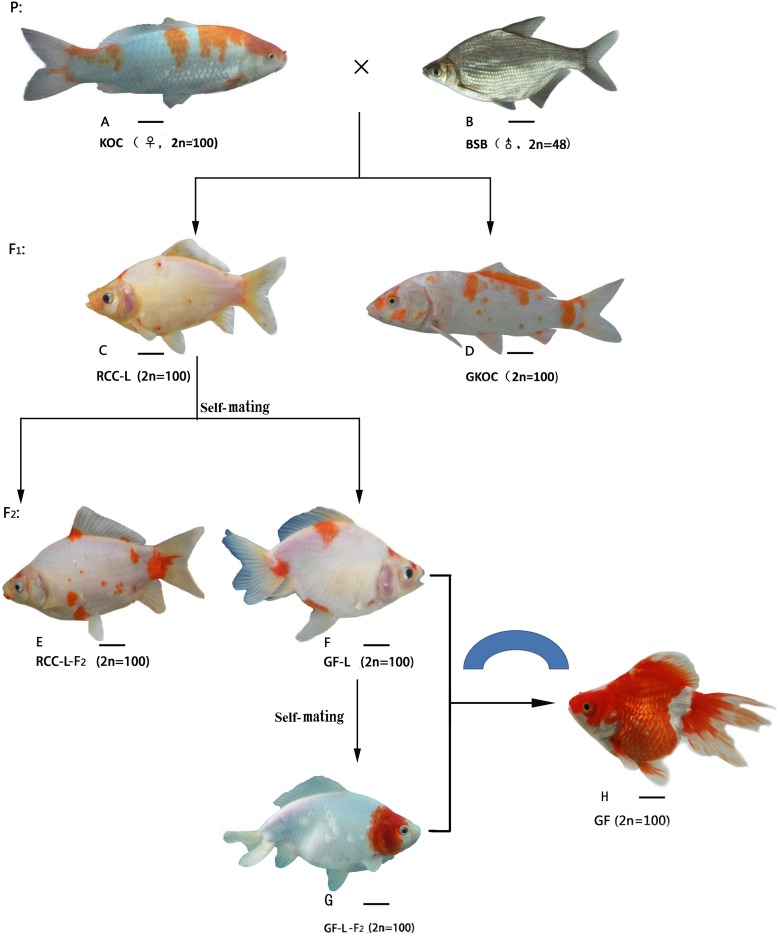
The formation procedure and the appearance of KOC, BSB, RCC-L, GKOC, GF-L, GF-L-F_2_ and GF. **(A)** KOC; **(B)** BSB; **(C)** RCC-L; **(D)** GKOC; **(E)** RCC-L; **(F)** GF-L; **(G)** GF-L-F_2_; and **(H)** GF. Bar = 3 cm.

The entire crossing procedure was shown in **Figure 1**. For each cross, 5,000 embryos were selected at random to determine fertilization (number of embryos at the gastrula stage/number of eggs × 100%), hatching (number of hatched fry/number of eggs × 100%), and survival (number of adulthood/number of eggs × 100%) rates. Simultaneously, self-mating of KOC and BSB were performed as controls. The hatched fry were transferred to a pond for further culture.

### Measurement of Morphological Traits

We randomly selected 60 1-year-old fish from each group (KOC, BSB, RCC-L, GF-L, RCC, and GF) for morphological examination. We measured whole length (WL), body length (BL), body height (BH), head length (HL), head height (HH), caudal peduncle length (CPL), and caudal peduncle height (CPH) of each fish (accurate to 0.1 cm). These values were then used to calculate the following ratios: BL/WL, BH/BL, HL/BL, HH/HL, CPH/CPL, and HH/BH. In addition, we recorded the number of lateral line scales, the number of scale rows above and below the lateral line, and the number of dorsal, anal, and pelvic fin rays. We used analysis of variance (ANOVA) (Osterlind et al., 2001) and multiple comparison tests (LSD method) (Williams and Abdi, 2010) to test for differences in each trait among the six types of fishes using SPSS Statistics 19.0 (IBM Corp., NY, United States). The values of the independent variables are expressed as the mean ± SD (Nigam and Turner, 1995).

### Preparation of Chromosome Spreads

To determine ploidy, chromosome preparation was carried out on the kidney tissues of 10 KOC, 10 BSB, 10 RCC-L, 10 GF-L, 10 RCC, and 10 GF at 1 year of age according to the procedures reported by Liu et al. (2001). We photographed 200 metaphase spreads from each sample to determine the chromosome number. Good-quality metaphase spreads were photographed and used for analysis of karyotypes. The chromosomal metaphase spreads were examined under an oil lens at a magnification of 3330×. Chromosomes were classified on the basis of their long-arm to short-arm ratios according to the reported standards (Levan et al., 1964).

### Microsatellite DNA Cloning and Sequencing

Total genomic DNA was isolated from whole blood collected from the caudal vein of 15 KOC, 15 BSB, 15 RCC-L, 15 RCC, 15 GF-L, and 15 GF using a standard phenol-chloroform procedure (Sambrook et al., 1989). DNA concentration and quality were assessed using agarose gel electrophoresis.

Three primer pairs (MFW1-F: 5′-AGCGGAACTCACTAAAC-3′, MFW1-R:5′-ACAGGCTTCCAGTAAAA-3′, MFW2-F: 5′-TTCATATCTCAGTGGCTT-3′, MFW2-R: 5′-ATCATTTATTCTTGTGGT-3′, MFW3-F: 5′-AGACAGCACTATCATTCC-3′, and MFW3-R: 5′-CCTAACATAAATAAACCCA-3′) were designed for the flanking regions of repeated (CA)n dinucleotide microsatellites based on RCC genome (Liu et al., 2016). The microsatellite loci were amplified and sequencing was performed as described by Liu et al. (2010). The genetic similarity was calculated as described by Nei and Li (1979).

### 5S rDNA, *chordinA* Cloning and Sequencing

One pair of primers (5SF: 5′-GCTATGCCCGATCTCGT CTGA-3′ and 5SR: 5′-CAGGTTGGTATGGCCGTAAGC-3′) (Sajdak et al., 1998) was designed and synthesized to amplify the 5S rDNA repeats directly from 10 KOC, 10 BSB, 10 RCC-L, 10 GF-L, 10 RCC, and 10 GF by PCR. One pair of primer (chordinA-F: 5′-TAACGCACAGATGCAGACGTGTG-3′ and chordinA-R, 5′-TGCTGTTCTCCTCAGAGCTGATGTAGG-3′) was designed and synthesized to amplify the chordin sequence directly from 10 RCC-L, 10 GF-L, 10 RCC, and 10 GF by PCR.

The PCR reactions and sequencing were performed as described by Qin et al. (2010) and Abe et al. (2014), respectively. Sequences were analyzed using BioEdit software (BioEdit version 7.0) (Hall, 1999).

### Mapping 5S rDNA to the Reference Genome

The genomes of CC, BSB, and RCC and their annotations were used as references for analyses of 5S rDNA obtained in this study. The above genomes were downloaded from the following websites:

(1) CC genome
ftp://ftp.ncbi.nlm.nih.gov/genomes/all/GCF/000/951/615/GCF_000951615.1_common_carp_genome (Xu et al., 2014)
(2) BSB genome:
https://www.ncbi.nlm.nih.gov/bioproject/?term=PRJNA343584 (Liu et al., 2017)
(3) RCC genome:
https://www.ncbi.nlm.nih.gov/bioproject/?term=PRJNA289059) (Liu et al., 2016)

We used BLASTN (*E*-value < = 10-5) to compare the sequences of 5S rDNA in RCC-L (203, 340, and 479 bp) and GF-L (168, 203, 340, and 495 bp) to the corresponding sequences of the genomes of CC, BSB, and RCC, respectively. Then we obtained the nucleotide similarities between the sequences of the above 5S rDNA and those from each of the genomes of CC, BSB, and RCC.

### Phylogenetic Analysis

Using Mega 5.1 (Tamura et al., 2011), the derived 5S rDNA coding gene sequences (120 bp) of these fragments were aligned from KOC, BSB, RCC-L, nature crucian carp (NCC), GF-L, RCC, and GF. Regions of sequences which were difficult to align were removed from the alignment. Gaps were also removed from the alignment. The maximum likelihood method implemented in the online software RAxML (Stamatakis, 2015) was used to construct a phylogenetic tree.

### Observation of Gonadal Structure

To observe the gonadal structure, we selected 10 10-month-old individuals of both RCC-L and GF-L. The gonads were fixed in Bouin’s solution for 24 h (Bancroft and Gamble, 2008; Ganjali and Ganjali, 2013), dehydrated using an ethanol gradient, and cleared in xylene. The gonadal sections were embedded in paraffin, cut at 7 μm, and stained with hematoxylin and eosin. The microstructure was observed and photographed using a Pixera Pro 600ES (Pixera Corporation, Santa Clara, CA, United States). We identified the gonadal development stages based on the standards for cyprinid fish (Liu, 1993).

## Results

### The Formation of RCC-L and GF-L

The crossing procedure to produce RCC-L and GF-L was outlined in **Figure 1**. In the first generation of KOC (♀) × BSB (♂), 99% RCC-L and 1% GKOC existed. The self-mating of RCC-L produced 98% RCC-L-F_2_ and 2% GF-L with twin tails. The self-mating of GF-L produced next generation of GF-L-F_2_ with twin tails.

### Fertilization, Hatching, and Survival Rates

The fertilized eggs of KOC (♀) × BSB (♂) showed high fertilization (90.5%) and hatching (80.3%) rates, but a low survival rate (35.6%). The self-mating of KOC resulted in a 95.6% fertilization rate, 85.3% hatching rate, and 80.7% survival rate, and the self- mating of BSB resulted in a 92.9% fertilization rate, 88.2% hatching rate, and 73.4% survival rate. In addition, the fertilization, hatching, and survival rates of RCC-L self-mating were 92.3, 85.8, and 76.3%, respectively.

### Morphological Traits and Feeding Habits

The morphological traits of KOC (**Figure 1A**), BSB (**Figure 1B**), RCC-L (**Figure 1C**), GKOC (**Figure 1D**), RCC-L-F_2_ (**Figure 1E**), GF-L (**Figure 1F**), GF-L-F_2_ (**Figure 1G**), and GF (**Figure 1H**) were shown in **Figure 1**. RCC-L and GF-L both exhibit broad phenotypic diversity. The individuals were generally distinguished from KOC by their body colors and shapes. One of the most recognizable features of the GF-L was the bifurcated tail.

**Table 1** presented the trait values for KOC, BSB, RCC-L, GF-L, RCC, and GF. Regarding the measured traits, RCC-L and their progeny had HH/BH values between and significantly different from those of KOC and BSB. In addition, RCC-L and their progeny had HL/BL values significantly greater than those of KOC and BSB and BL/WL values significantly lower (*P* < 0.05) than those of KOC and BSB. The HH/HL value in RCC-L was lower (*P* < 0.05) than that in either KOC or BSB and was markedly higher (*P* < 0.05) than that in GF-L or KOC or BSB. RCC-L exhibited BH/BL value similar to that of BSB but different from that of KOC. The BH/BL in GF-L was higher (*P* < 0.05) than that in KOC or BSB. The CPH/CPL value of RCC-L was between that of KOC and that of BSB and markedly different from both, whereas CPH/CPL in GF-L was lower than that in KOC or BSB. The RCC-L and RCC had similar CPH/CPL values. The HH/HL value of GF-L was significantly higher (*P* < 0.05) than that of GF. In other measurable traits (BL/WL, BH/BL, CPH/CPL, and HH/BH), there was no significant difference (*P* > 0.05) between GF and GF-L.

**Table 1:** The phenotypes including the measurable traits (the average ratios of body length to whole length (BL/WL), body height to body length (BH/BL), head length to body length (HL/BL), head height to head length (HH/HL), caudal peduncle height to caudal peduncle length (CPH/CPL), and head height to body height (HH/BH), and the countable traits (number of lateral scales, number of dorsal fins, number of abdominal fins, number of anal fins in RCC-L, and their progeny and their parents).

Phenotypes	Types of fish				
	
	KOC	BSB	RCC-L	GF-L	RCC	GF
BL/WL	0.86 ± 0.01	0.84 ± 0.04	0.82 ± 0.05	0.65 ± 0.01	0.82 ± 0.02	0.60 ± 0.02
BH/BL	0.38 ± 0.01	0.43 ± 0.04	0.43 ± 0.03	0.69 ± 0.04	0.41 ± 0.02	0.72 ± 0.06
HL/BL	0.25 ± 0.02	0.21 ± 0.02	0.26 ± 0.02	0.36 ± 0.01	0.31 ± 0.03	0.45 ± 0.04
HH/HL	0.96 ± 0.03	0.88 ± 0.02	0.61 ± 0.06	1.13 ± 0.03	0.88 ± 0.06	0.90 ± 0.06
CPH/CPL	0.80 ± 0.09	0.93 ± 0.01	0.83 ± 0.20	0.15 ± 0.03	0.93 ± 0.11	0.18 ± 0.05
HH/BH	0.63 ± 0.05	0.49 ± 0.01	0.61 ± 0.06	0.60 ± 0.08	0.65 ± 0.02	0.70 ± 0.06
No. of lateral scales	35.5 ± 0.71	50.94 ± 0.94	26.65 ± 1.14	29.67 ± 1.52	28.60 ± 1.14	28.90 ± 0.08
No. of upper lateral scales	7.50 ± 0.71	9.67 ± 0.49	6.90 ± 0.31	7.33 ± 0.58	5.40 ± 0.55	5.40 ± 0.51
No. of lower lateral scales	5.50 ± 0.69	10.05 ± 0.64	5.40 ± 0.50	6.33 ± 0.58	5.36 ± 0.89	6.50 ± 0.25
No. of dorsal fins	*III* + 20.5 ± 0.68	*III* +8.67 ± 0.49	*III* + 17.9 ± 1.02	*III* +14.67 ± 0.90	*III* + 18.60 ± 0.55	*III* +14.67 ± 1.02
No. of abdominal fins	11.5 ± 0.65	9.06 ± 0.64	8.60 ± 0.68	9.33 ± 0.65	7.80 ± 0.84	8.60 ± 0.24
No. of anal fins	*III* + 9.5 ± 0.62	*III* + 25.89 ± 0.68	*III* + 7.15 ± 0.59	*III* + 7.67 ± 0.58	*III* + 6.42 ± 0.55	*III* + 6.6 ± 0.24


Regarding the countable traits, all values (i.e., number of lateral scales, number of upper lateral scales, number of lower lateral scales, number of abdominal fins, and number of anal fins) except the number of dorsal fins in RCC-L and GF-L were significantly lower than those in KOC and BSB (*P* < 0.05). For number of dorsal fins, the RCC-L and GF-L had values intermediate between KOC and BSB. RCC and RCC-L presented no significant differences (*P* > 0.05). All countable traits had no significant difference (*P* > 0.05) in GF-L and GF.

Regarding feeding habits, RCC-L, RCC, GF-L, and GF similar to BSB were herbivorous.

### Chromosome Numbers and Karyotypes

**FIGURE 2:**
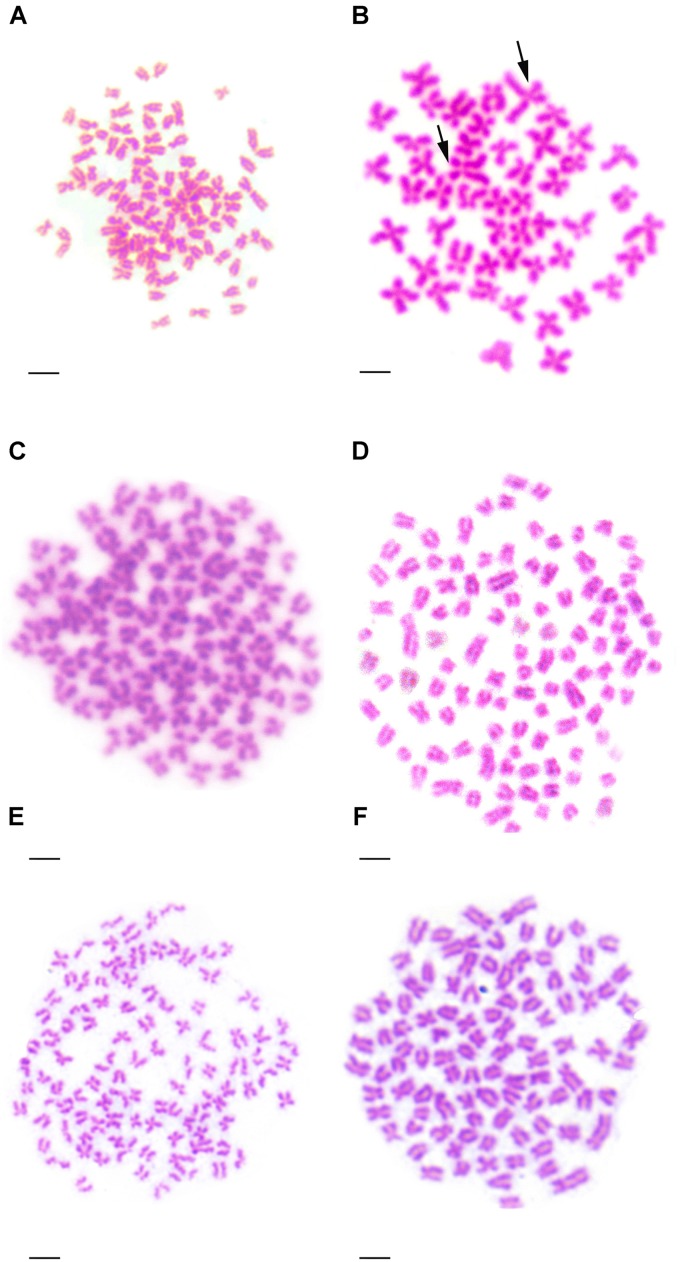
Chromosome spreads at metaphase in KOC, BSB, RCC-L, GF-L, GF, and RCC. **(A)** The 100 chromosomes of KOC. **(B)** The 48 chromosomes of BSB in which a pair of the largest submetacentric chromosomes (black arrows) is indicated. **(C)** The 100 chromosomes of RCC-L in which the largest submetacentric chromosome was not found. **(D)** The 100 chromosomes of GF-L in which the largest submetacentric chromosomes were not found. **(E)** The 100 chromosomes of GF in which the largest submetacentric chromosomes were not found. **(F)** The 100 chromosomes of RCC in which the largest submetacentric chromosomes was not found. Bar = 3 μm.

**FIGURE 3:**
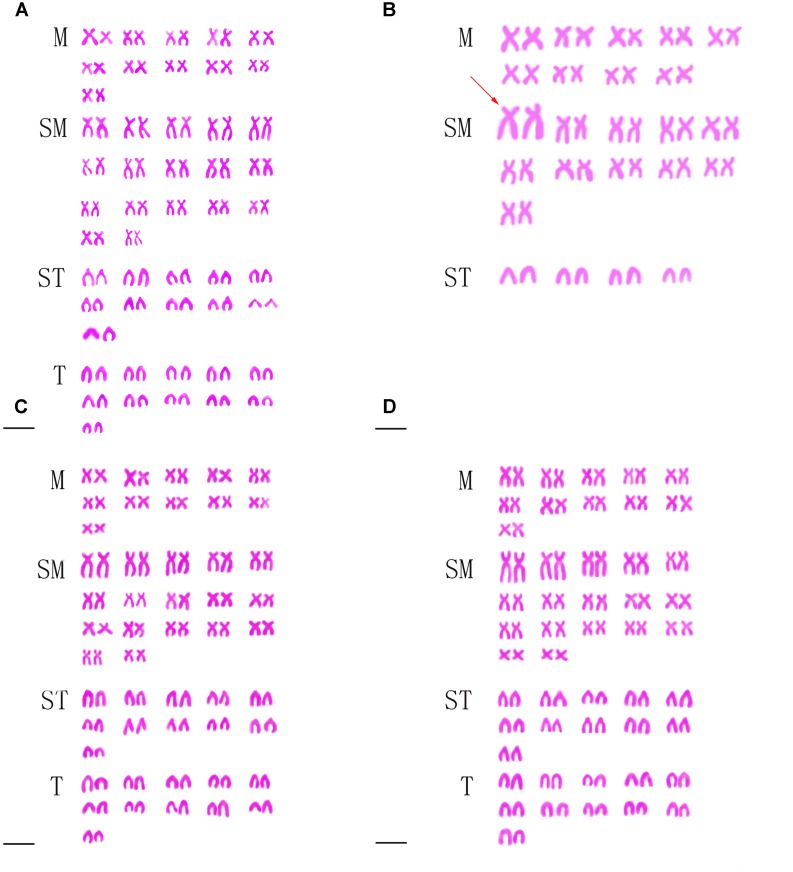
Karyotypes of KOC, BSB, RCC-L, and GF-L. **(A)** The karyotype of KOC is 22m + 34sm + 22st + 22t. **(B)** The karyotype of BSB is 18m + 22sm + 8st. **(C)** The karyotype of RCC-L is 22m + 34sm + 22st + 22t. **(D)** The karyotype of GF-L is 22m + 34sm + 22st + 22t. Bar = 3 μm. The red arrow indicates the two largest submetacentric chromosomes.

**Table 2** presented the distribution of chromosome number in KOC, BSB, RCC-L, GF-L, RCC, and GF. Among KOC, 91.0% of the chromosomal metaphase spreads exhibited 100 chromosomes (**Table 2**), indicating that KOC was diploid with 100 chromosomes (**Figure 2A**) with a karyotype of 22m + 34sm + 22st + 22t (**Figure 3A**) (m, the chromosome with the cross in the median region; sm, submedian region, st, subterminal region; t, terminal region). Among BSB, 88.0% of the spreads exhibited 48 chromosomes (**Table 2**), indicating that BSB was diploid with 48 chromosomes and a karyotype of 18m + 22sm + 8st (**Figure 3B**). A large pair of submetacentric chromosomes was observed in BSB, which was used as a chromosomal marker to identify this species (**Figure 2B**). Among KOC chromosomes, there was no large submetacentric chromosome. Among RCC-L, 87.5% of the chromosomal metaphase spreads had 100 chromosomes (**Figure 2C**) with a karyotype 22m + 34sm + 22st + 22t (**Figure 3C**), indicating that RCC-L was diploid. Among GF-L, 90.0% of the chromosomal metaphase spreads had 100 chromosomes (**Figure 2D**) with a karyotype of 22m + 34sm + 22st + 22t (**Figure 3D**), indicating that GF-L was diploid. Among GF, 90.0% of the metaphases had 100 chromosomes (**Figure 2E**). Among RCC, 92.5% of the metaphases had 100 chromosomes (**Figure 2F**). Unlike BSB, RCC-L, and GF-L exhibited no large submetacentric chromosome. The above results indicated that the typical number of chromosomes in RCC-L, RCC, GF-L, and GF was 100.

**Table 2:** Chromosome numbers in KOC, BSB, RCC-L, GF-L, RCC, and GF.

		Distribution of chromosome number											
		
Fish type	No. of metaphase	<48	48	<100	100	<148	148	<150	150	<198	198	<200	200
KOC	200			18	182								
BSB	200	24	176										
RCC-L	200			25	175								
GF-L	200			20	180								
RCC	200			15	185								
GF	200			20	180								


### Microsatellite DNA

Three pairs of microsatellite primers (MFW1, MFW2, and MFW3) were used to analyze the genomic traits in RCC-L, GF-L, KOC, BSB, RCC, and GF. With the MFW1 primers, only one band with 150 bp was amplified in RCC-L, whereas two bands with 150 and 130 bp were amplified in RCC (**Supplementary Figure S1**), suggesting that RCC-L and RCC can be identified by these primers.

**FIGURE 4:**
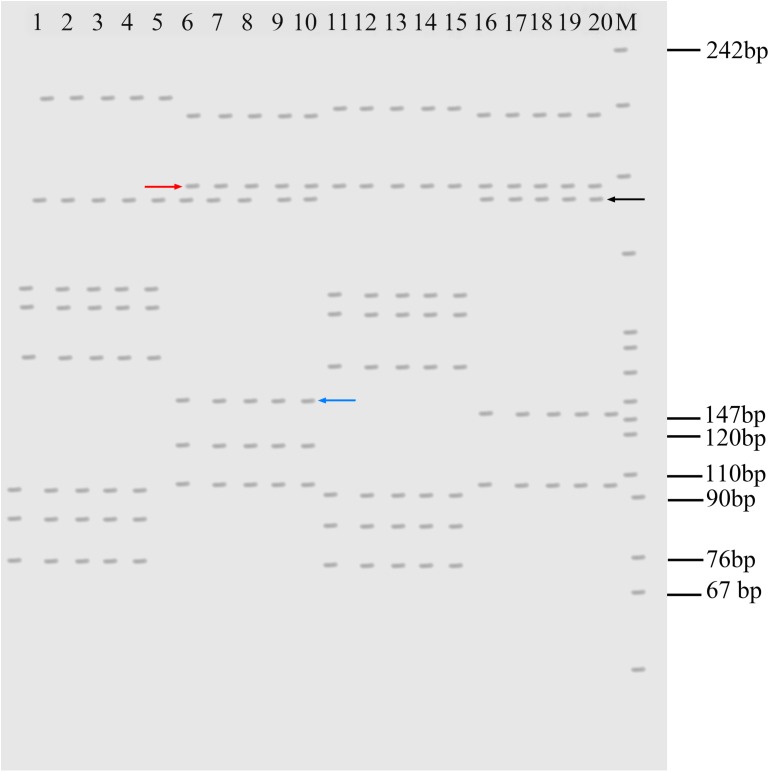
Electrophotogram of microsatellite DNA patterns produced by the primer MFW2 in KOC, RCC-L, BSB, and GF-L. Lanes 1–5 represent KOC. Lanes 6–10 represent RCC-L. Lanes 11–15 represent BSB. Lanes 16–20 represent GF-L. The black arrow indicates the DNA bands derived from KOC. RCC-L and GF-L commonly had this type of band, but BSB did not have this band. The red arrow indicates the DNA bands derived from BSB. RCC-L and GF-L commonly had this type of band, but KOC did not have this band. The blue arrow shows the band found only in RCC-L. M represents the pBR322 DNA/Mspl Marker.

With the MFW2 primer, KOC and BSB were detected by yielding different microsatellite DNA patterns (**Figure 4**). RCC-L exhibited some DNA fragments similar to those of KOC (**Figure 4**, black arrow), suggesting that RCC-L inherited those DNA fragments from KOC. Furthermore, RCC-L had some DNA fragments (**Figure 4**, red arrow) similar to those presented by BSB, showing that RCC-L also inherited some DNA fragments from BSB. Interestingly, a new DNA fragment (**Figure 4**, blue arrow) that was not observed in either KOC or BSB was observed in both RCC-L and GF-L, suggesting DNA variation in RCC-L that was inherited from RCC-L to GF-L.

With the MFW3 primer, the genotypic similarity of RCC-L and RCC was 95.00%, whereas the genotypic similarities of GF to GF-L was 98.30%, showing the RCC-L and RCC as well as GF and GF-L had high similarity.

### 5S rDNA and *chordinA*

**FIGURE 5:**
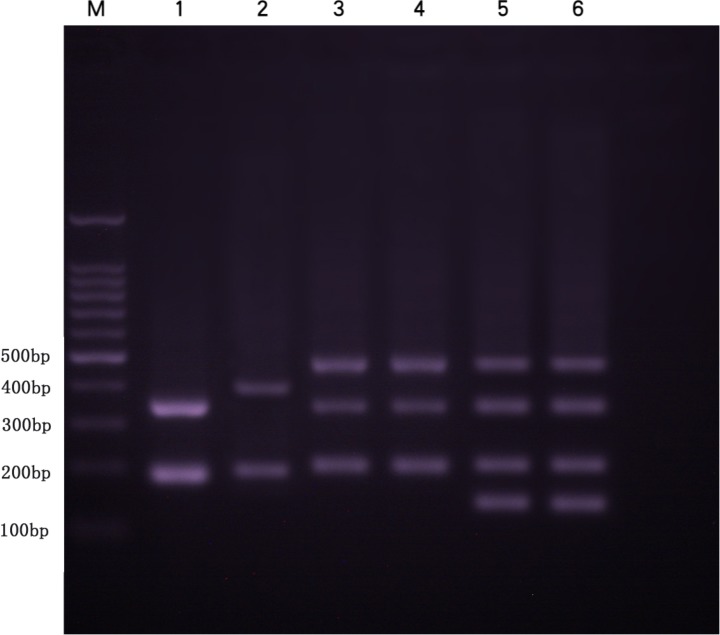
DNA bands (5S rDNA) amplified by the primer pair 5SF-5SR in BSB, KOC, RCC-L, RCC, GF, and GF-L. Lane 1, two DNA fragments (approximately 200 and 360 bp) found in BSB. Lane 2, two DNA fragments (approximately 200 and 400 bp) found in KOC. Lane 3, three DNA fragments (approximately 200, 340, and 500 bp) found in RCC-L. Lane 4, three DNA fragments (approximately 200, 340, and 500 bp) found in RCC. Lane 5, four DNA fragments (approximately 160, 200, 340, and 500 bp) found in GF-L. Lane 6, four DNA fragments (approximately 160, 200, 340, and 500 bp) found in GF. M represents DNA ladder markers (100 bp increments).

Several DNA fragments were amplified from KOC, BSB, RCC-L, GF-L, RCC, and GF using 5S rDNA primer pair. These PCR fragments generated distinct agarose gel electrophoresis band patterns. There were two fragments (approximately 200 and 400 bp) in KOC (MH909573 and MH909574) and two fragments (approximately 180 and 360 bp) in BSB (GQ485554 and KT824058.1), three fragments (approximately 200, 340, and 500 bp) in RCC-L, four fragments (approximately 160, 200, 340 and 500 bp) in GF-L, three fragments (approximately 200, 340 and 500 bp) in RCC (GQ485555, GQ485556, and GQ485557), and four fragments (approximately 160, 200, 340, and 500 bp) in GF (**Figure 5**) (GU188688, GU188687, GU188689, and GU188690). Based on the BLASTN analyses, all fragments from KOC, BSB, RCC-L, GF-L, GF, and RCC were confirmed as 5S rDNA repeat units (**Table 3**).

**Table 3:** Results of 5S rDNA DNA fragments by PCR and sequenced clone number.

		PCR DNA fragments and sequenced clone number			
		
Fish type	Total number of sequenced clones	∼200 bp	∼340 bp	∼400 bp	∼500 bp
KOC	40	20 sequenced clones of 203 bp	Absent	20 sequenced clones of 406 bp	Absent
BSB	40	30 sequenced clones of 188 bp	10 sequenced clones of 367 bp	Absent	Absent
RCC-L	40	20 sequenced clones of 203 bp	10 sequenced clones of 340 bp	Absent	10 sequenced clones of 495 bp
GF-L	40	10 sequenced clones of 168 bp; 10 sequenced clones of 203 bp	10 sequence clones of 340 bp	Absent	10 sequenced clones of 495 bp
RCC	40	20 sequenced clones of 203 bp	10 sequenced clones of 340 bp	Absent	10 sequenced clones of 495 bp
GF	40	15 sequenced clones of 168 bp; 5 sequenced clones of 203 bp	10 sequenced clones of 340 bp	Absent	10 sequenced clones of 495 bp


The sequences of 5S rDNA units cloned in this study contained a coding region (5′-99 bp and 3′-21 bp) and a mid-region consisting of distinct NTS sequences. In BSB, only monomeric 5S rDNA (designated class I: 188 bp) was characterized by one NTS type (designated NTS-I: 68 bp). In KOC, only monomeric 5S rDNA (designated class II: 203 bp) was characterized by one NTS type (designated NTS-II: 83 bp). In RCC-L, there were three monomeric 5S rDNA classes (designated class II: 203 bp; class III: 340 bp; and class IV: 495 bp) that were characterized by three NTS types (designated NTS-II: 83 bp, NTS-III: 220 bp, and NTS-IV: 375 bp). In GF-L and GF, there were four monomeric 5S rDNA classes (designated class V: 168 bp; class II: 203 bp; class III: 340 bp; and class IV: 495 bp) (**Supplementary Figure S2**) which were characterized by four NTS types (designated NTS-V:48 bp; NTS-II:83 bp; NTS-III: 220 bp; and NTS-IV: 375 bp) (**Supplementary Figure S3**). In RCC, there were also three monomeric 5S rDNA classes (class I, class II, and class IV), which had three NTS sequences (NTS-II, NTS-III, and NTS-IV), respectively.

The KOC, RCC-L, GF-L, RCC, and GF all had 203 bp DNA fragments in 5S rDNA. This fragment exhibited high similarities among the different kinds of fishes. The similarities between KOC and RCC-L, KOC and GF-L, KOC and RCC, and KOC and GF were 83.70, 84.20, 84.25, and 85.20%, respectively. The similarities between RCC-L and GF-L, RCC-L and RCC, RCC-L and GF were 92.10, 93.50, and 95.50%, respectively. The similarities between GF-L and RCC, and GF-L and GF were 92.60 and 93.50%, respectively. The similarities between RCC and GF was 96.00%. Among them, the highest similarity was between RCC and GF, which reached 96.00% (**Supplementary Figure S4** and **Table 4**).

**Table 4:** The percentages of nucleotide identity of 5S rDNA (class II)sequences in KOC, RCC-L, GF-L, RCC, and GF.

Ratio of different fishes involved	Similarity (%)
KOC: RCC-L	83.70
KOC: GF-L	84.20
KOC: RCC	84.25
KOC: GF	85.20
RCC-L: GF-L	92.10
RCC-L: RCC	93.50
RCC-L: GF	92.10
GF-L: RCC	92.60
GF-L: GF	93.50
RCC: GF	96.00


Comparative analyses of the NTS sequences indicated several base substitutions or insertions-deletions between RCC-L and RCC. The NTS-I sequences of RCC-L and RCC were highly similar (with 97.5% average similarity). The NTS-II sequence of RCC-L showed an average 90.4% similarity to that of RCC. The sequence comparison of NTS-III between RCC-L and RCC indicated 93.05% identity. The sequence comparisons of RCC-L and RCC among classes II, III, and IV revealed 99.5% identity for class II, 91.4% identity for class III, and 91.9% identity for class IV, revealing that the sequences of those DNA fragment in the RCC-L were highly homologous to those of RCC (**Supplementary Figure S5**).

The 5S rDNA coding regions (CDS) of KOC, BSB, RCC-L, GF-L, GF, and RCC exhibited similarities of 97.5, 97.5, 97.5, 96.6, and 95.0%, respectively. The sequence comparison of 5S rDNA CDS between RCC-L and RCC resulted in 98.3% identity, suggesting that RCC-L and RCC were derived from similar parents. The sequence comparison of 5S rDNA CDS between GF-L and GF resulted in 97.5% identity, showing that GF-L and GF were also derived from the similar parents. The sequence comparison of 5S rDNA CDS among GF-L, KOC, BSB, RCC-L, and RCC presented a 91.7% identity between GF-L and KOC, a 90.9% identity between GF-L and BSB, a 92.5% identity between GF-L and RCC-L, and a 92.5% identity between GF-L and RCC (**Table 5** and **Supplementary Figure S6**).

**Table 5:** The percentages of nucleotide identity of 5S rDNA (coding region) sequences in KOC, BSB, RCC-L, GF-L, RCC, and GF.

Ratio of different fishes involved	Similarity (%)
KOC: BSB	99.10
KOC: RCC-L	97.50
KOC: GF-L	91.70
KOC: RCC	97.50
KOC: GF	93.30
BSB: RCC-L	96.60
BSB: GF-L	90.90
BSB: RCC	96.60
BSB: GF	92.50
RCC-L: GF-L	92.50
RCC-L: RCC	98.30
RCC-L: GF	94.10
GF-L: RCC	92.50
GF-L: GF	95.00
RCC: GF	95.09


**FIGURE 6:**
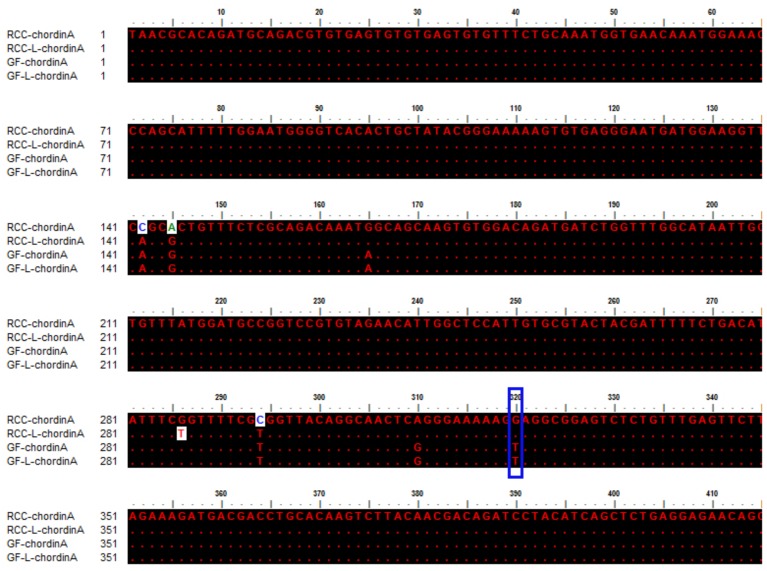
Sequence comparison of *chordinA* in RCC, RCC-L, GF-L, and GF. The blue box represents the mutation base regarding the formation of twin tails. The dots represent the same base.

The sequences of *chordinA* in GF-L, GF, RCC-L, and RCC were compared (MH898971, MH898974, MH898972, and MH898970), which indicated that the 320th location base was T in GF-L and GF, whereas the 320th location base in RCC-L and RCC was G, respectively (**Figure 6**). This mutation (G-T) showed that RCC-L and GF-L formed excellent fish lineage for studying gene variation and function. The present results were in accordance with a previous study in which the position base mutation (G-T) was found to possibly contribute to the occurrence of a twin tail in GF (Abe et al., 2014, 2016). In addition, compared with RCC, we found that there were some base site mutations (137th position:C-A; 140th position:A-G; 294th position:C-T) in the RCC-L sequence, indicating that there was variation in the RCC-L genome (**Figure 6**).

### The Sequences of 5S rDNA in RCC-L and GF-L Aligned With the Genomes of Related Species

The sequences of 5S rDNA in RCC-L (203, 340, and 479 bp) and GF-L (168, 203, 340, and 495 bp) (MH898963, MH898964, MH898965, MH898966, MH898967, MH898968, and MH898969) were mapped to the corresponding sequences in the CC, BSB, and RCC genomes as references, respectively. The results were shown in **Table 6**.

**Table 6:** The percentages of nucleotide identity of 5S rDNA sequences in RCC-Land GF-L compared with the genomes of related species.

	Common carp genome			Blunt snout bream genome			Red crucian carp genome		
			
Amplification fragment	Alignment location	Alignment length	Alignment similarity	Alignment location	Alignment length	Alignment similarity	Alignment location	Alignment length	Alignment similarity
RCC-L 203 bp	NW_017538106.1 416752-416555	199	98.03	scaffold270 293264-293360	98	48.28	scaffold_12 30497-30699	203	100.00
RCC-L 340 bp	NW_017545642.1 24976-24658	338	99.41	scaffold270 293264-293358	95	27.94	repeat_87021 13-349	337	91.12
RCC-L 479 bp	NC_031730.1 15592863-15592771	93	19.42	scaffold556 113490-113398	93	19.42	scaffold_12 55954-56362	411	85.80
GF-L 168 bp	NW_017544418.1 23526-23620	95	56.55	scaffold270 293264-293359	97	57.74	repeat_125478 1404-1571	168	100.00
GF-L 203 bp	NW_017538537.1 142565-142724	160	78.82	scaffold1773 23157-23232	76	37.44	scaffold_12 30497-30699	203	100.00
GF-L 340 bp	NW_017545642.1 24976-24658	338	99.41	scaffold556 113490-113392	99	29.12	repeat_87021 13-349	340	100.00
GF-L 495 bp	NC_031730.1 15592863-15592769	95	19.19	scaffold556 113490-113392	99	20.00	scaffold_12 55954-56362	423	85.45


As for RCC-L, CC, and BSB, the nucleotide similarities of the sequences of 5S rDNA (203, 340, and 479 bp) of RCC-L to CC (genome) were 98.03, 99.41, and 19.42%, respectively, whereas those similarities of RCC-L to BSB (genome) were 48.28, 27.94, and 19.42%, respectively, showing that the average similarity (72.29%) of RCC-L to CC was obviously higher than that (31.88%) of RCC-L to BSB. Because KOC is a variety of CC, we conclude that the similarity of RCC-L to KOC is higher than that of RCC-L to BSB.

For GF-L, CC, and BSB, the nucleotide similarities of the sequences of 5S rDNA (168, 203, 340, and 495 bp) of GF-L to CC (genome) were 56.55, 78.82, 99.41, and 19.19%, respectively, whereas those similarities of GF-L to BSB (genome) were 57.74, 37.44, 29.12, and 20.00%, respectively, indicating that the average similarity (63.67%) of GF-L to CC was obviously higher than that (36.08%) of GF-L to BSB. Because KOC is a variety of CC, we conclude that the similarity of GF-L to KOC is higher than that of GF-L to BSB.

Regarding RCC-L and RCC, the nucleotide similarities of the sequences of 5S rDNA (203, 340, and 479 bp) of RCC-L to RCC (genome) were 100.00, 91.12, and 85.80%, respectively, whereas those similarities of RCC (5S rDNA) to RCC (genome) were 100.00, 100.00, and 100.00%, respectively, showing genomic DNA variation in RCC-L.

Regarding GF-L, GF, and RCC, the nucleotide similarities of the sequences of 5S rDNA (168, 203, 340, and 495 bp) of GF-L to RCC (genome) were 100.00, 100.00, 100.00, and 85.45%, whereas those similarities of GF to RCC (genome) were 100.00, 100.00, 94.12, and 86.00%, respectively, showing the average similarity (96.36%) of GF-L to RCC was almost equal to that (95.03%) of GF to RCC.

The map of relationships between the 5S rDNA sequences and the corresponding sequences in the genomes of CC, BSB, and RCC as references were shown in **Supplementary Figure S7**.

### Phylogenetic Relationships

**FIGURE 7:**
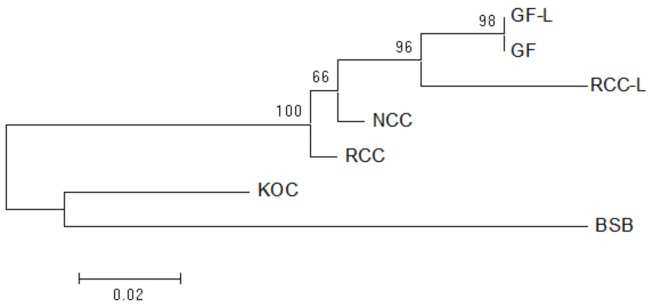
Maximum likelihood tree inferred from 5S rDNA sequences. This tree illustrates the phylogenetic relationships among 5S rDNA sequences in KOC, BSB, RCC-L, NCC, GF-L, RCC, and GF. The numbers at the branch nodes indicate the bootstrap percentage.

Using the NJ method in Mega software, the phylogenetic tree of GF-L, GF, RCC-L, NCC, RCC, KOC, and BSB was constructed. The largest tree span appeared between GF-L and BSB, and the smallest tree span between in GF-L and GF. GF-L and GF formed a sister group. The tree distance between GF and KOC was smaller than that of GF and BSB. (**Figure 7**).

### Gonadal Microstructure of KOC, BSB, RCC-L, and GF-L

**FIGURE 8:**
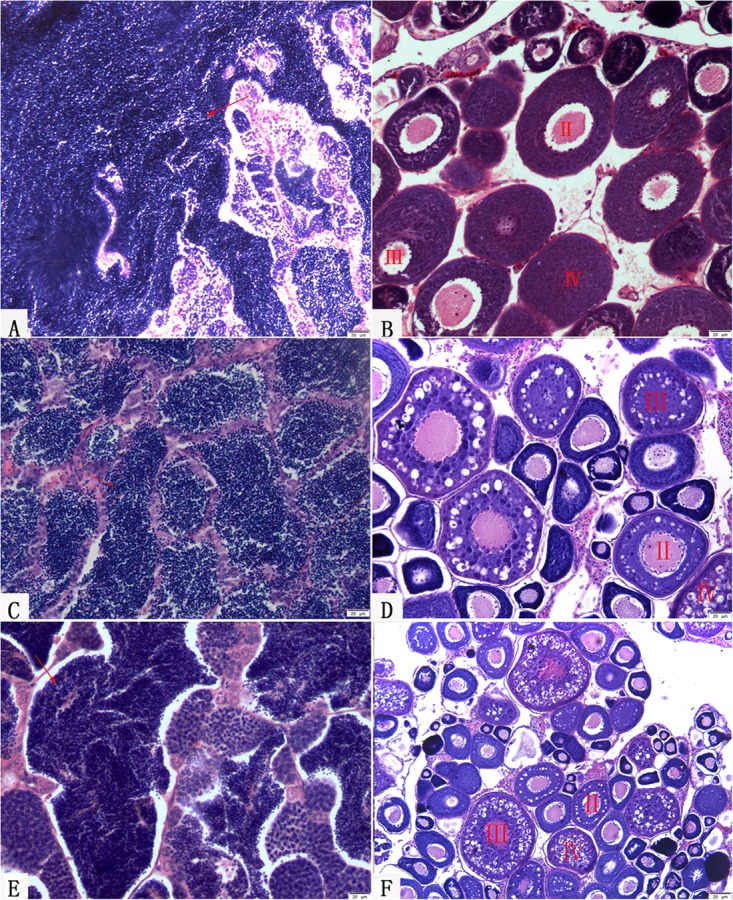
Gonadal structure of BSB, KOC, RCC-L, and GF-L. **(A)** Mature testis of BSB containing mature spermatozoa, spermatids and spermatogonia. **(B)** Mature ovary of KOC including II-phase, III-phase, and IV-phase oocytes. **(C)** Mature testis of RCC-L containing mature spermatozoa, spermatids and spermatogonia. **(D)** Mature ovary of RCC-L including II-phase, III-phase, and IV-phase oocytes. **(E)** Mature testis of GF-L containing mature spermatozoa, spermatids and spermatogonia. **(F)** Mature ovary of GF-L including II-phase, III-phase, and IV-phase oocytes. Bar = 20 μm.

Two-year-old BSB and 2-year-old KOC were able to produce normal mature gamete (**Figures 8A,B**; Liu et al., 2013; Wen et al., 2013). Moreover, 1-year-old RCC-L and 1-year-old GF-L were able to produce normal mature gametes. We stripped white semen from 10-month-old males RCC-L and GF-L and mature ova from 10-month-old females RCC-L and GF-L. In the testes of 1-year-old RCC-L and GF-L, we observed numerous mature spermatozoa, spermatids, and spermatogonia in the seminiferous tubules (**Figures 8C,E**). Observation of the gonadal tissue sections revealed that the ovaries of 8-month-old RCC-L and GF-L females were at stages III and IV, indicating that RCC-L and GF-L were fertile (**Figures 8D,F**).

## Discussion

### Origin of Goldfish

Extensive comparative studies of GF and crucian carp found that they not only exhibited similar phenotypes and fertility in the hybrids of GF and crucian carp (Fu, 2016), but also shared the same embryonic developmental processes and chromosome number (2*n* = 100) (Changcheng, 1988; Tsai et al., 2013). GF and crucian carp were generally believed to be closely related, and were classified within the same species, but belonged to different varieties. Based on many biochemical and molecular phylogenetic analyses, including isozyme amplification, muscle protein electrophoresis, serotype identification, RAPD, and mitochondrial DNA analyses (Komiyama et al., 2009), it was concluded that GF evolved from crucian carp. However, the direct evidence is lacking.

In this study, the distant hybridization of KOC (2*n* = 100, ♀) and BSB (2*n* = 48, ♂) produced RCC-L (2*n* = 100) in F_1_; subsequent self-mating of RCC-L produced 2% GF-L (2*n* = 100) with double tailfins; self-mating of GF-L generated GF-L-F_2_, which provided clear evidence for the pathway of the formation of the GF as shown as KOC– (KOC as a variety of CC)-color crucian carp–GF (**Figure 1**).

GF-L and RCC-L were showed to be homodiploids mainly derived from the genome of KOC with some DNA fragments from BSB (**Figures 1**–**7**; **Tables 1**–**5**). GF-L and RCC-L presented obviously different traits from KOC and BSB (**Table 1**). For example, in terms of phenotypes, GF-L and RCC-L had obvious different HH/BH, HL/BL, BL/WL, and HH/HL values, and different number of lateral scales, number of abdominal fins, and number of anal fins from their parents. In terms of reproductive traits, the GF-L and RCC-L had different sexual mature age (1-year) from that (2-year) of KOC and BSB (**Figure 8**), further indicating that GF-L and RCC-L were potentially new species with the same chromosomal number (2*n* = 100) as their maternal parent (KOC), but with different phenotypes and genotypes from their parents.

In terms of genotypes, GF-L and RCC-L showed different microsatellite DNA patterns and different 5S rDNA sequences from those of KOC and BSB (**Figure 4**, **Table 4** and **Supplementary Figure S4**), suggesting that DNA variation occurred in GF-L and RCC-L. The presence of multicopy of 5S rDNA, which was probably due to gene conversion resulting from the parental genome (Holliday, 1964; Sun et al., 1989; Martins and Galetti, 1999), showed further evidence for the DNA variation occurring in GF-L and RCC-L.

By comparing the *chordinA* sequences in GF-L, GF, RCC-L, and RCC, we found that the 320th location base in GF-L and GF was T, whereas the 320th location base in RCC-L and RCC was G (**Figure 6**). This mutation (G-T) showed that RCC-L and GF-L formed an excellent fish lineage for studying gene variation and function. The present results were in accordance with a previous study in which the position base mutation (G-T) was found to possibly contribute to the occurrence of twin tails in GF (Abe et al., 2014, 2016). The mitochondrial genome of RCC-L also presented a large number of variations (unpublished data).

The results of mapping the sequences of 5S rDNA in GF-L and RCC-L to each of the genomes of CC and BSB as references provided further evidence that RCC-L and GF-L were derived from both KOC and BSB. KOC is a variety of CC. The genome of KOC is a always the same as that of CC. The average similarity of each of GF-L and RCC-L to CC was obviously higher than that to BSB, supporting that the genome of both RCC-L and GF-L is mainly inherited from KOC, but with some DNA fragments from BSB.

The comparative analyses of the phenotypes and genotypes, as well as the reproductive traits between GF-L and GF, and between RCC-L and RCC, indicated that GF-L was very similar to GF, and RCC-L was very similar to RCC. For example, the morphological characteristics of GF-L and GF showed no significant difference (*P* > 0.05) in BL/WL, BH/BL, and CPH/CPL. The morphological characteristics of RCC-L and RCC showed no significant difference (*P* > 0.05) in BL/WL, BH/BL, HL/BL, HH/BH, and the number of lower lateral scales (**Table 2**). Regarding the genotypes, the similarities regarding the sequences of microsatellite DNA between GF-L and GF, and between RCC-L and RCC, were 95.00 and 98.30%, respectively, indicating that their similarities in genotypes were very high. On the other hand, the chromosomal numbers in GF-L, GF, RCC-L, and RCC were all 100 (**Figure 2**). For the reproductive traits, the age of sexual maturity was 1 year in GF-L, GF, RCC-L, and RCC (**Figure 8**).

The analyses of the phylogenetic tree based on the 5S rDNA sequences, showed that GF-L and GF were located in the same group and were close to RCC-L and RCC (**Figure 7**), providing further evidence that the pathway of RCC-GF existed. On the other hand, GF-L, GF, RCC-L, and RCC were closer to KOC than BSB (**Figure 7**), supporting the existence of a KOC-RCC-GF pathway.

Although most of the characteristics of RCC-L were similar to those of RCC, some differences were found between them. For instance, RCC-L presented unique microsatellite bands which were not found in its parents and RCC (**Supplementary Figure S1**). The results of mapping the sequences of the 5S rDNA of RCC-L to the RCC genome showed genomic variation in RCC-L (**Table 6** and **Supplementary Table S1**). These results indicated that genomic incompatibilities and genomic shock arose from distant hybridization and resulted in genomic DNA changes in RCC-L. These genomic variations might explain why RCC-L could easily reproduce GF-L with many phenotypic changes including the presence of two-tails, whereas it was difficult for RCC to reproduce GF. The RCC-L had been subjected to genomic incompatibilities and genomic shock due to distant hybridization and was in the “plastic” stage that was prone to produce genomic variations and novel traits.

Based on the presence of the GF-L derived from RCC-L self-mating, we concluded that GF was probably derived from RCC self-mating. Despite the low frequency (2%) of the formation of GF-L, we established the persistent RCC-L and GF-L and GF-L-F_2_ lineages as the neodiploid population, providing new evidence regarding the origins of GF via the KOC–RCC–GF pathway, indicating that interspecific hybridization has the potential to form new species, which is importance to species evolution research.

### Significance of GF-L

As a new type of goldfish-like fish, GF-L and GF-L-F_2_ presented very beautiful phenotypes, especially (**Figure 1**) those with twin tails and white bodies accompanied by red spots. These phenotypes were quite different from any other GF, indicating that the GF-L lineage had great potential in the ornamental market. On the other hand, GF-L possessed greater genomic DNA variations, which could easily result in phenotypic changes. GF-L has been used as a new fish resource to cross with other GFs to produce a series of new types of GFs with beautiful phenotypes. The formation of GF-L was very important to both evolutionary biology and fish genetic breeding.

## Author Contributions

SL conceived and designed the study. YW and CY contributed to the experimental work, performed most of the statistical analyses, and wrote the manuscript. QQ, JS, and MZ designed the primers and performed the bioinformatics analyses. KL and YH collected the experimental materials. MT and CZ collected the photographs. All authors read and approved the final manuscript.

## Conflict of Interest Statement

The authors declare that the research was conducted in the absence of any commercial or financial relationships that could be construed as a potential conflict of interest.
