# Supplementary material for: The Formation of the Goldfish-Like Fish Derived From Hybridization of Female Koi Carp × Male Blunt Snout Bream
Source: Front Genet. 2018 Oct 10;9:437. doi: 10.3389/fgene.2018.00437 (PMC6194320; doi:10.3389/fgene.2018.00437)
Supplement: Supplementary file 1 [file Table_1.DOCX]

**Supplementary table and table legends**

**Supplementary Table 1 The percentages of nucleotide identity of 5S rDNA sequences in RCC and GF compared with the related species’ genomes**

|  | Common carp genome | | | Blunt snout bream genome | | | Red crucian carp genome | | |
| --- | --- | --- | --- | --- | --- | --- | --- | --- | --- |
| Amplification fragment | Alignment Location | Alignment length | Alignment similarity | Alignment Location | Alignment length | Alignment similarity | Alignment Location | Alignment length | Alignment similarity |
| RCC 203 bp | NW_017538106.1  416752-416555 | 199 | 98.03 | scaffold270  293264-293360 | 98 | 48.28 | scaffold_12  30497-30699 | 203 | 100.00 |
| RCC 340 bp | NW_017545642.1  24658-24976 | 338 | 99.41 | scaffold556  113392-113490 | 99 | 29.12 | repeat_87021  13-349 | 337 | 99.12 |
| RCC 479bp | NC_031730.1  15592771-15592863 | 93 | 19.42 | scaffold110  1908863- 1908959 | 98 | 20.46 | scaffold_12  55954-56362 | 423 | 88.31 |
| GF 168 bp | NW_017544418.1  23526-23620 | 95 | 56.55 | scaffold270  293264-293359 | 97 | 57.74 | repeat_125478  1404-1571 | 168 | 100.00 |
| GF 203bp | NW_017537762.1  275936-276133 | 199 | 98.03 | scaffold270  293264-293360 | 97 | 47.78 | scaffold_12  30497-30699 | 203 | 100.00 |
| GF 340bp | NC_031730.1  15592769-15592863 | 95 | 27.94 | scaffold556  28744-28838 | 95 | 27.94 | scaffold_17228  1348917-1349230 | 320 | 94.12 |
| GF 495bp | NW_017545642.1  24761-24976 | 233 | 47.07 | scaffold270  293264-293360 | 97 | 19.60 | scaffold_12  55954-56362 | 430 | 86.87 |

**Supplementary figures and their legends**

**Supplementary Figure S1. Electrophotogram of microsatellite DNA patterns produced by the primer MFW1 in RCC-L and RCC**

Lanes 1-9 represent RCC-L. Lanes 10- 19 represented RCC. Red arrow indicates the special DNA bands only found in RCC. M represents the pBR322 DNA/Mspl Marker.

**Supplementary Figure S2. The 5S rDNA sequence comparison between GF-L and GF**

**Supplementary Figure S3. Representative sequences of 5S rDNA classⅠ, Ⅱ, Ⅲ and Ⅳ from GF-L. Complete 5S coding regions are shaded**

**Supplementary Figure S4. Comparison of the ClassⅡsequences in KOC, RCC-L, GF-L, RCC and GF.**

**The dots represent the same base.**

**Supplementary Figure S5**. **The 5S rDNA sequence comparison between RCC-L and RCC**

**Supplementary Figure S6. Comparison of 5S rDNA coding region sequences (120 bp) in KOC, BSB, RCC-L, GF-L, RCC and GF**

**Supplementary Figure S7. Map of relationships between the 5S rDNA sequences and the corresponding sequences in the reference genome (CC genome, BSB genome and RCC genome)**

A: Map of relationships between the reference genome and RCC-L. B: Map of relationships between the reference genome and GF-L. C: Map of relationships between the reference genome and RCC. D: Map of relationships between the reference genome and GF.

Figure S1


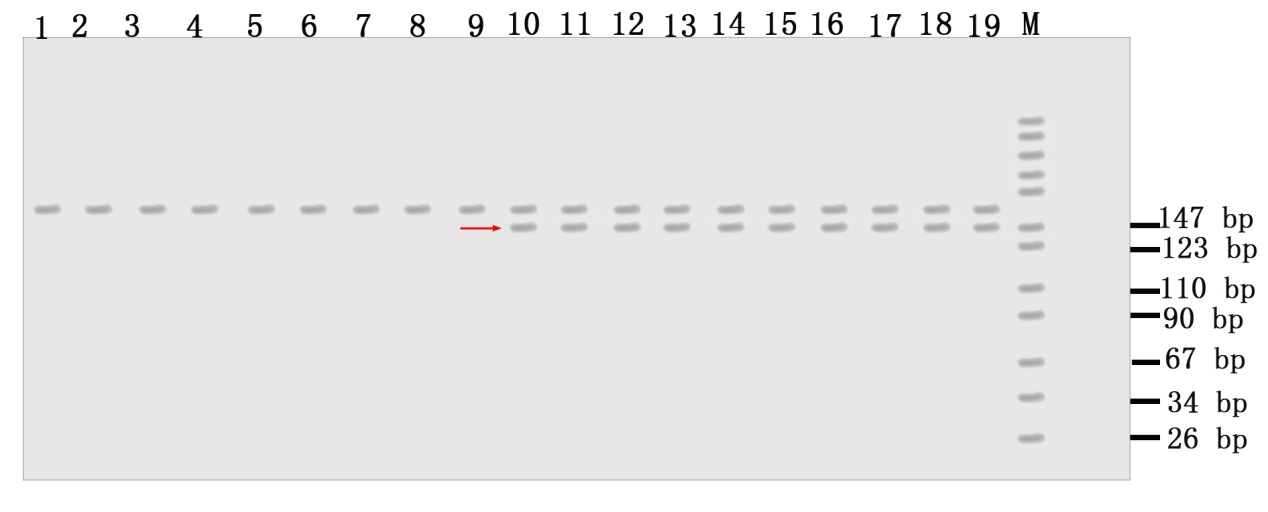


Figure S2


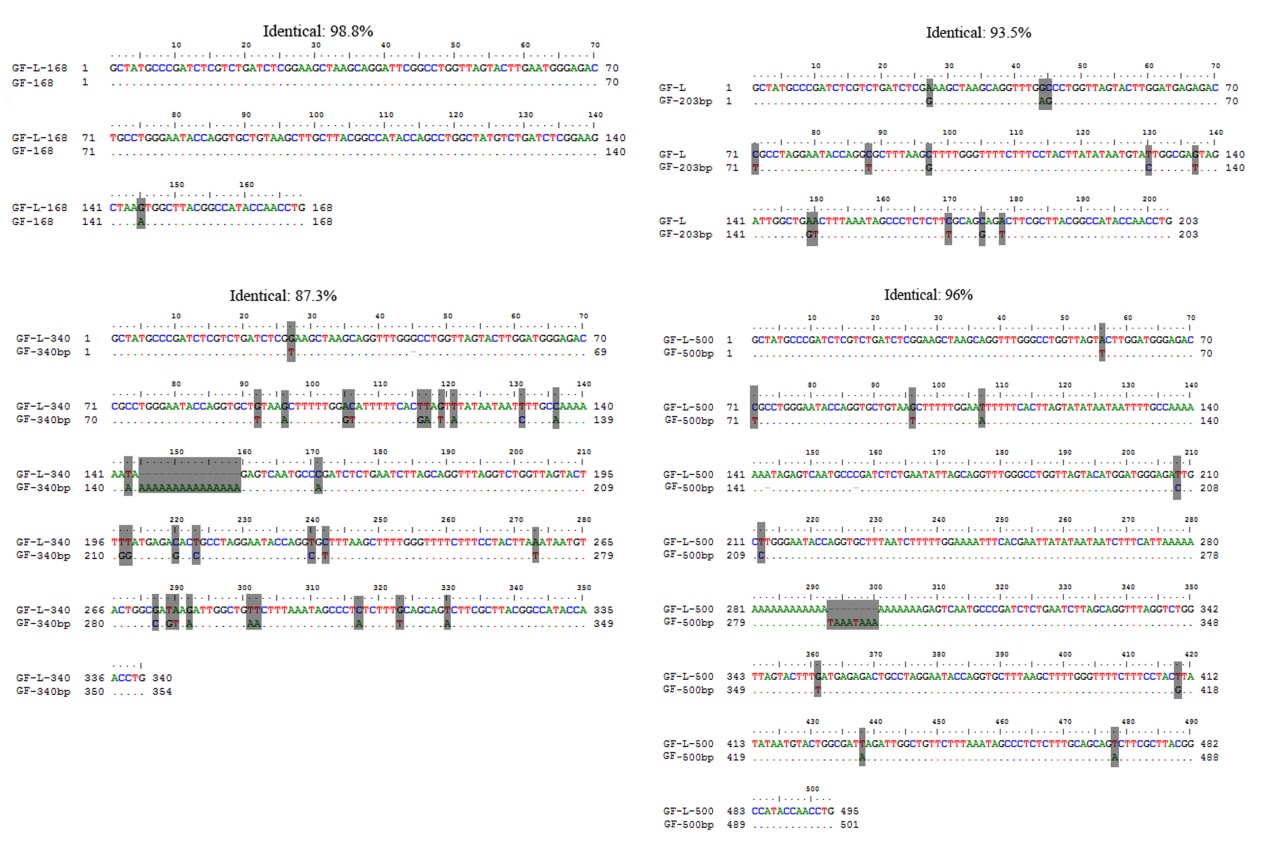


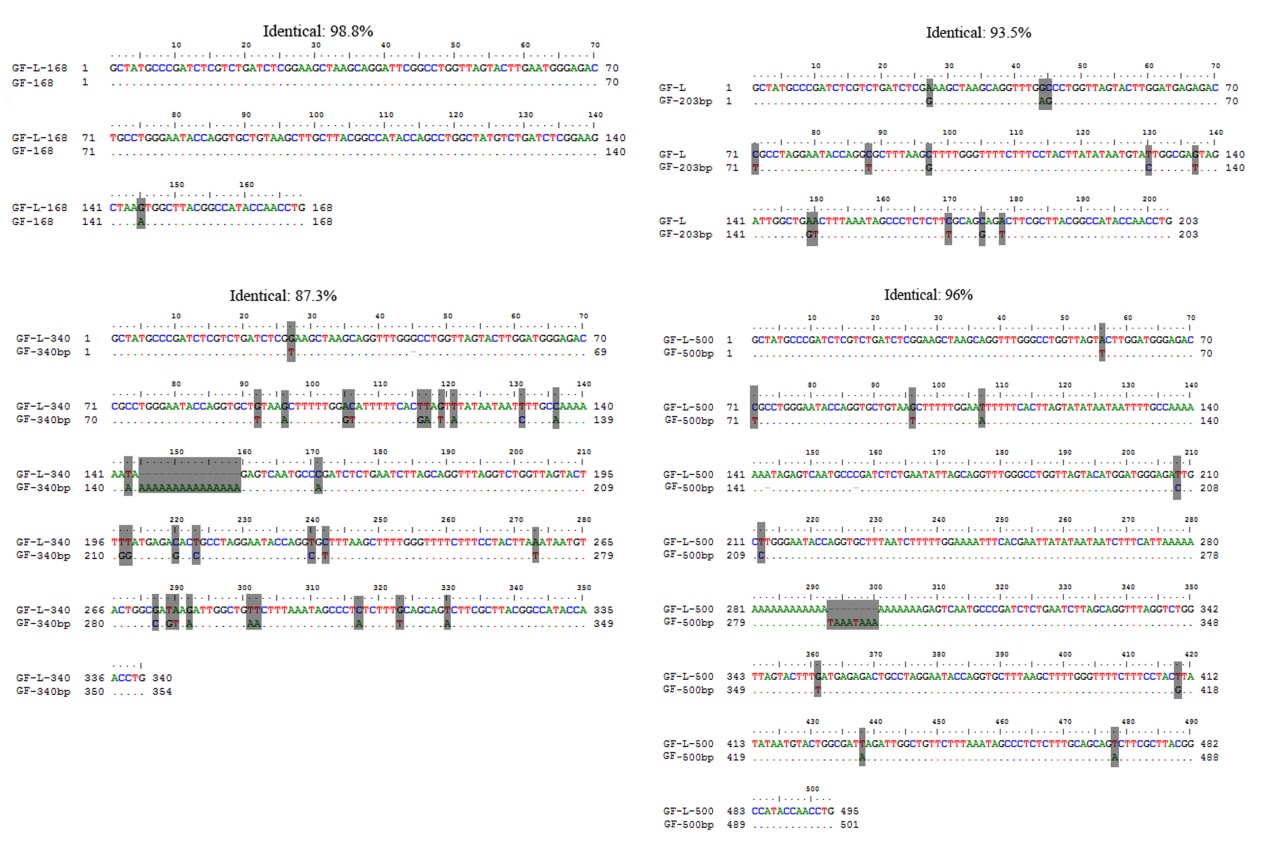


Figure S3


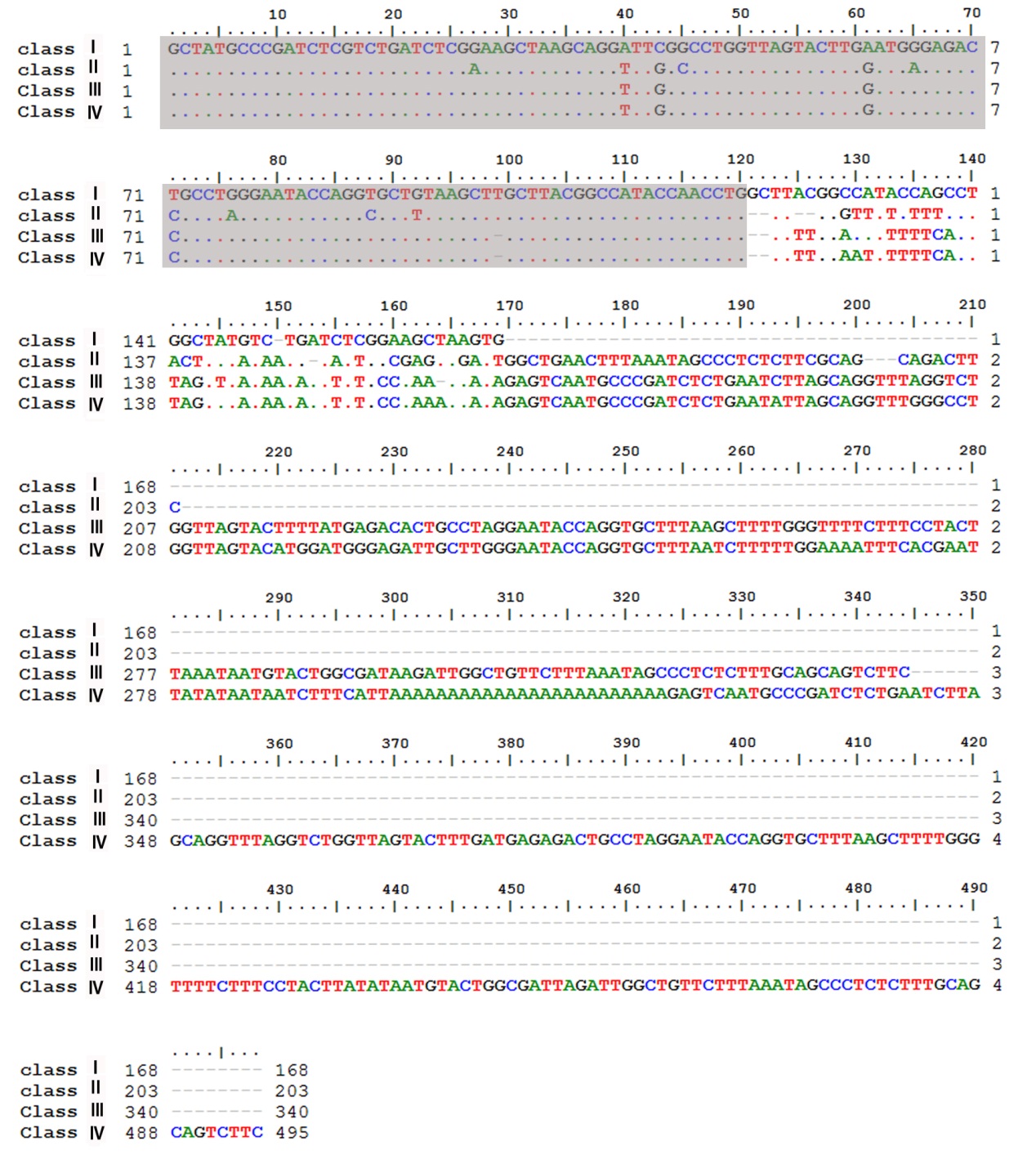


Figure S4


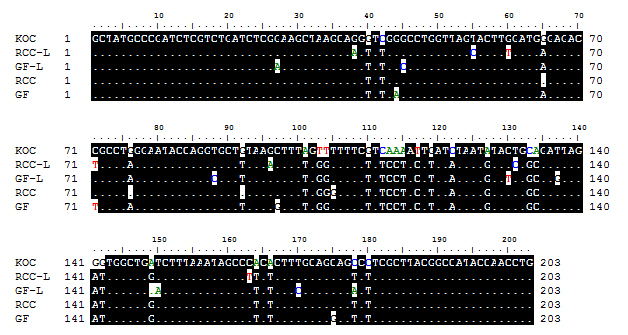


Figure S5


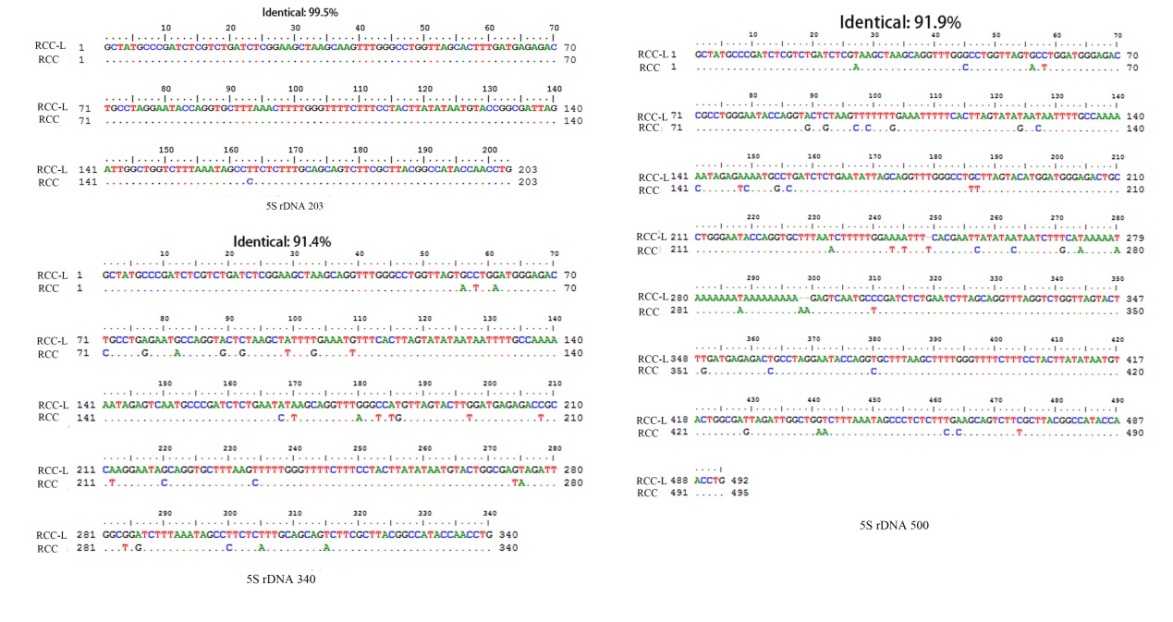


Figure S6


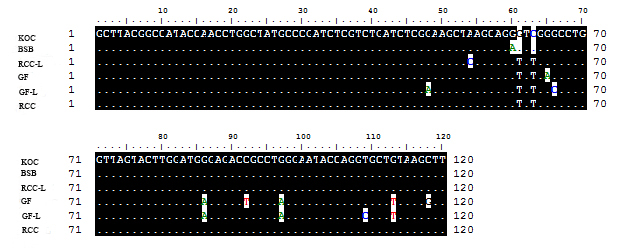


Figure S7


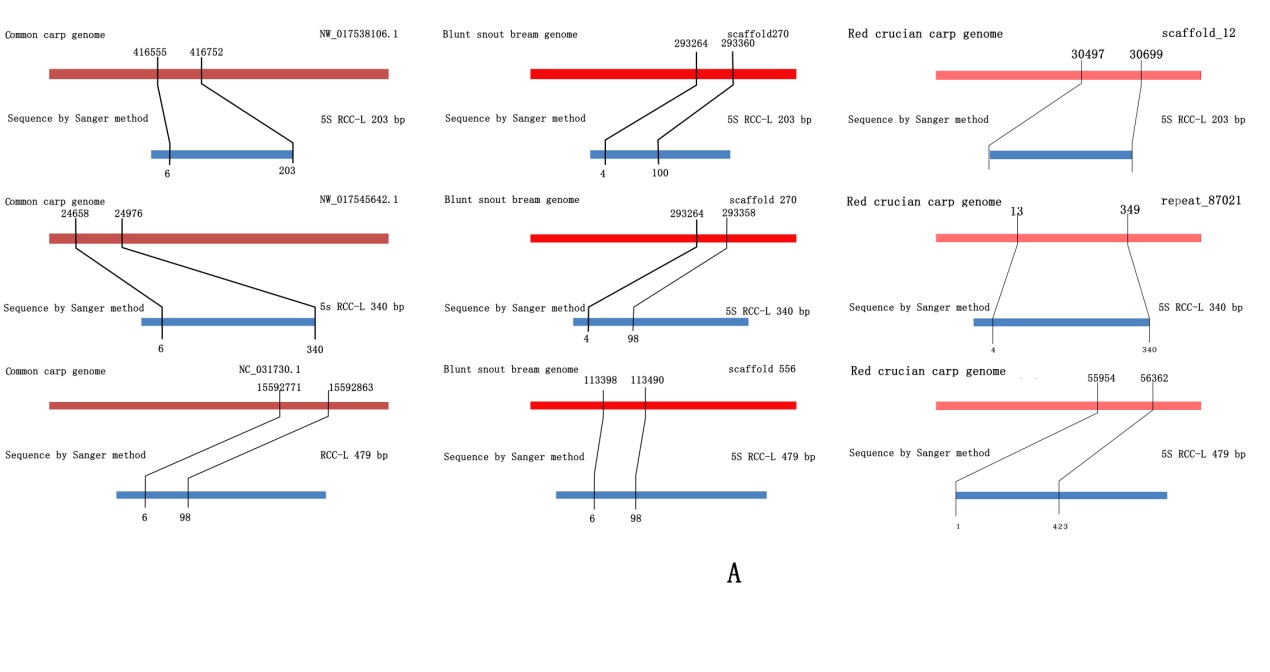

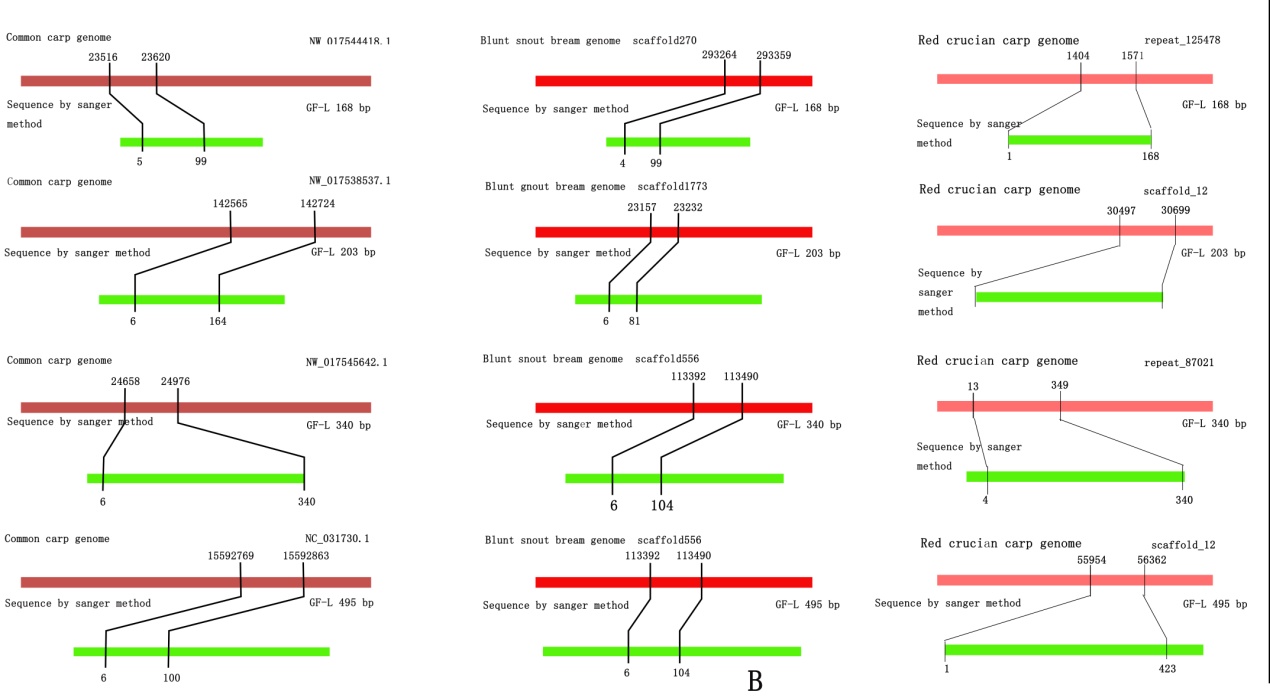


B

A


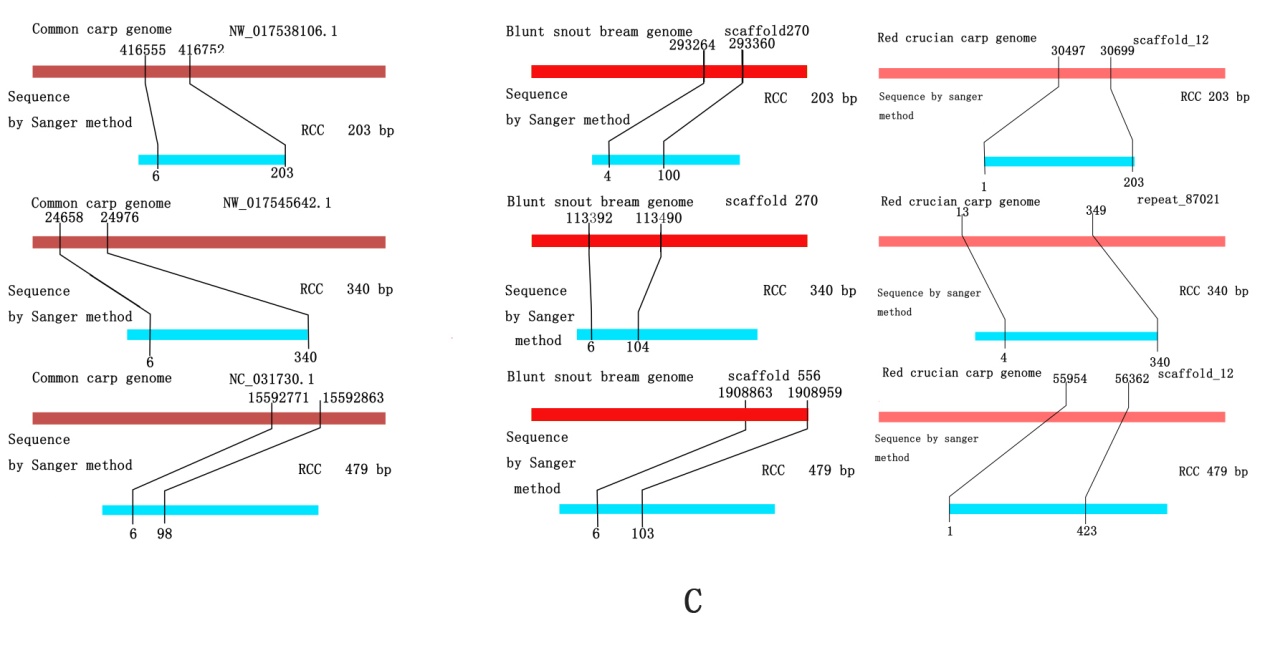

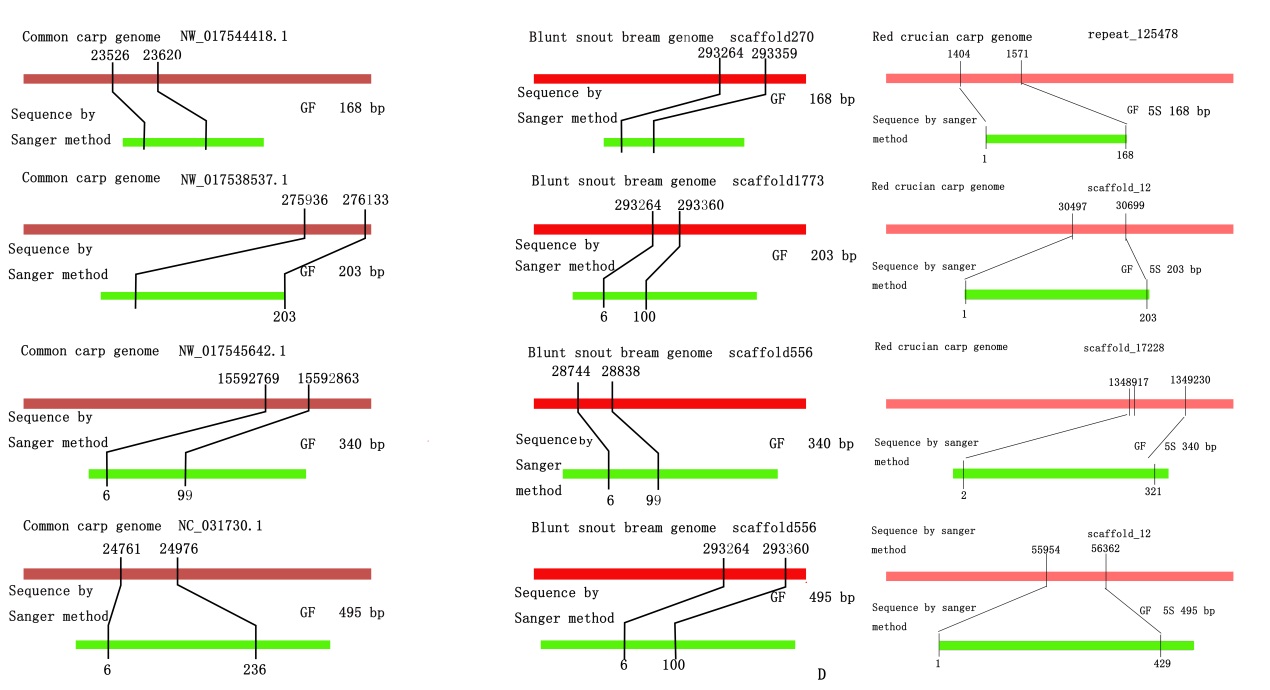


C

D
